# Ruptured Amebic Liver Abscess

**DOI:** 10.1155/1990/41597

**Published:** 1990

**Authors:** Toyokazu Kawano, Yuji Ichiyoshi, Susumu Nagasaki, Tomohiro Toda, Yoshikazu Minamisono, Yukinori Okazaki, Takashi Kanematsu, Keizo Sugimachi

**Affiliations:** 1 Department of Surgery The Institute of Gastroenterology of Hofu 14-33 Eki-Minami Hofu 747 Japan; 2 Department of Internal Medicine I Faculty of Medicine Yamaguchi University Ube 755 Japan; 3 Department of Surgery II Faculty of Medicine Kyushu University 3-1-1 Maidashi, Higashi-ku Fukuoka 812 Japan

## Abstract

We treated two patients with a ruptured amebic liver abscess. The diagnosis was made at a relatively early stage and treatment was successful for one patient, but an accurate diagnosis of liver abscess was not made and invasive extraintestinal amebiasis led to multiple organ failure and to death for the other. Neither patient had been outside of Japan, and both were heterosexual. The origins of *Entamoeba histolytica* infection could not be determined. Though the mortality rate is high in cases of ruptured amebic liver abscess, appropriate management can lead to a good prognosis.


*HPB Surgery*, 1990, Vol. 3, pp. 21–28
Reprints available directly from the publisher
Photocopying permitted by license only

© 1990 Harwood Academic Publishers GmbH
Printed in the United Kingdom

### RUPTURED AMEBIC LIVER ABSCESS

TOYOKAZU KAWANO, YUJI ICHIYOSHI, SUSUMU NAGASAKI, TOMOHIRO TODA and YOSHIKAZU MINAMISONO
*Department of Surgery, The Institute of Gastroenterology of Hofu 14–33 Eki-Minami, Hofu 747, Japan*

YUKINORI OKAZAKI
*Department of Internal Medicine I, Faculty of Medicine, Yamaguchi University Ube 755, Japan*

TAKASHI KANEMATSU and KEIZO SUGIMACHI
*Department of Surgery II, Faculty of Medicine, Kyushu University 3-1-1 Maidashi, Higashi-ku, Fukuoka 812, Japan*

*(Received 10 January 1990)*

We treated two patients with a ruptured amebic liver abscess. The diagnosis was made at a relatively early stage and treatment was successful for one patient, but an accurate diagnosis of liver abscess was not made and invasive extraintestinal amebiasis led to multiple organ failure and to death for the other. Neither patient had been outside of Japan, and both were heterosexual. The origins of *Entamoeba histolytica* infection could not be determined. Though the mortality rate is high in cases of ruptured amebic liver abscess, appropriate management can lead to a good prognosis.

KEY WORDS: Amebiasis, liver abscess, rupture

### INTRODUCTION

*Entamoeba histolytica* is the only species of parasitic protozoa with the potential for producing human amebiasis, transmitted by ingesting cysts present in contaminated food and water. In Japan, amebiasis had decreased since the 1950's as the result of improved sanitation, and in 1978, only 6 patients with amebiasis were registered at the Ministry of Health and Welfare of Japan. However, the number of amebiasis patients registered in 1985 was 137. This increase may be mainly due to the increasing number of high-risk people those who travel to areas in the world where *Entamoeba histolytica* infection is endemic, and possibly to increases in homosexuality[1,2]. Amebic liver abscess is a common extraintestinal manifestation of amebiasis, and the incidence of liver abscess in patients of amebic colitis was reported to be in the range of 3–40%[3,4]. Since peritonitis secondary to amebic liver abscess is usually fatal[5,6], early recognition and treatment of this entity is most important to save the patient's life[7,8]. In case of a ruptured amebic liver abscess, drainage of abdominal and/or pleural cavity as well as administration of antiamebic agents and other clinical support should be carried out immediately.

Correspondence to: T. Kawano.

### CASE REPORTS

### *Case 1*

A 32-year-old taxi driver was admitted to another hospital on April 15 in 1985, with complaints of pain in the right upper quadrant. He had never been abroad and his past medical and social history were not contributory. Abdominal ultrasonography (US) on admission showed an enlarged gall bladder but without stones. He underwent laparotomy under a tentative diagnosis of acute cholecystitis. The gall bladder was acutely inflamed and externl cholecystostomy was done. Postoperatively, he ran a fever, and US and chest X-ray revealed a fluid collection in the right pleural cavity and right subphrenic space. Each cavity was drained on the 6th and 13th postoperative days. He was then referred to the Department of Surgery II, Kyushu University Hospital on May 1, 1985. On admission, he was in distress and drowsy; body temperature 37.0°C, pulse rate 105 beats/min, blood pressure 150/70 mmHg, and respiratory rate 23 breaths/min. He was jaundiced, and there was evidence of erythema in the anterior chest wall and edema in the lower extremities. Moist rales were present in bilateral lung fields. The abdomen was distended but soft, and bowel sounds were weak.

Laboratory data were as follows; hemoglobin level 6.0 g/dl, white blood cell count 42,600/mm$^3$. Serum chemistry showed albumin 2.0 g/dl, total bilirubin 22.2 mg/dl, direct bilirubin 18.8 g/dl, alkaline phosphatase 539 U/1, aspartate transaminase (AST) 464 U/1, alanine transaminase (ALT) 155 U/1, lactate dehydrogenase (LDH) 539 U/1, cholesterol 30 mg/dl, triglyceride 64 mg/dl, blood urea nitrogen 160 mg/dl, creatinine 4.1 mg/dl, and uric acid 14.6 mg/dl.

He underwent re-laparotomy on May 1 with a tentative diagnosis of a right subphrenic abscess. However, this was proved to be a ruptured liver abscess for which drainage was carried out. Culture of the fluid taken from the abscess cavity showed it to be aseptic. Histological examination of the debris from the abscess cavity revealed trophozoites of *E. histolytica*. Indirect hemaggulutination (IHA) test for *E. histolytica* was positive in the serum of the patient. Metronidazole (1000 mg/day) was prescribed by mouth or rectum, but he died about 2 weeks after admission. Autopsy revealed an amebic liver abscess, hepato-bronchial fistula, pyothorax, panperitonitis and a cecal ulcer.

### *Case 2*

A 71-year-old man who cleaned buildings was admitted to the Institute of Gastroenterology of Hofu on February 6, 1988, with complaints of high fever and headache of 3 days duration. His past medical history included partial gastrectomy for duodenal ulcer at the age of 46, followed by surgical interventions 3 times for small bowel obstruction. He and his family had never been abroad.

On admission, he was not in distress; body temperature 38.6°C, pulse rate 80 beats/min, blood pressure 122/70 mmHg, and respiratory rate 20 breaths/min. Breath sounds were clear. The abdomen was flat and soft, the liver was not palpable, but ther was a slight tenderness in the right upper quadrant. There was no splenomegaly and the bowel sounds were slightly decreased.

Laboratory data on admission included a hemoglobin of 11.5 g/dl and white blood cell count of 15,100/mm$^3$. Alkaline phosphatase was 16.4 U (normal value

2.6–10 U), choline esterase was 0.51 U (normal value 0.55–1.20 U), but the values of other liver function tests were within normal limits.

The high fever continued, and right upper quadrant became increasingly tender. US showed a large multilocular low echogenic lesion in the right lobe of the liver. In computed tomography (CT), a multilocular low density mass about 9 cm in the greatest diameter was seen in the right lobe of the liver (Figure 1). These findings were consistent with a diagnosis of a liver abscess. Percutaneous transhepatic abscess drainage (PTAD) was performed on February 9, and about 200 ml of dark brown fluid was obtained. Culture of this material showed no evidence of bacterial contamination. Since body temperature remained at around 40°C, contrast rentogenography through a PTAD tube was done and there was evidence of rupture of the liver abscess (Figure 2). The stool was bloody and examination of pus and stool showed *E. histolytica* trophozoites phagocyting red blood cells (Figure 3). The IHA

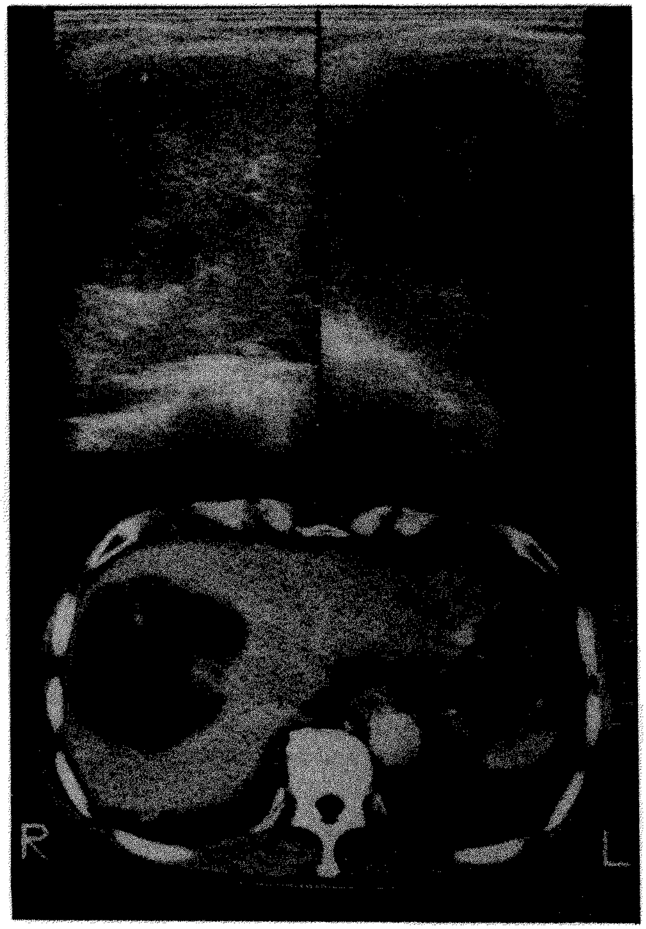

**Figure 1** Abdominal ultrasonography (top) and computed tomography of amebic liver abscess in the Case 2 patient. In the right lobe of the liver, a large multilocular low echogenic lesion was evident and a multilocular low density mass about 9 cm in diameter was revealed by computed tomography.

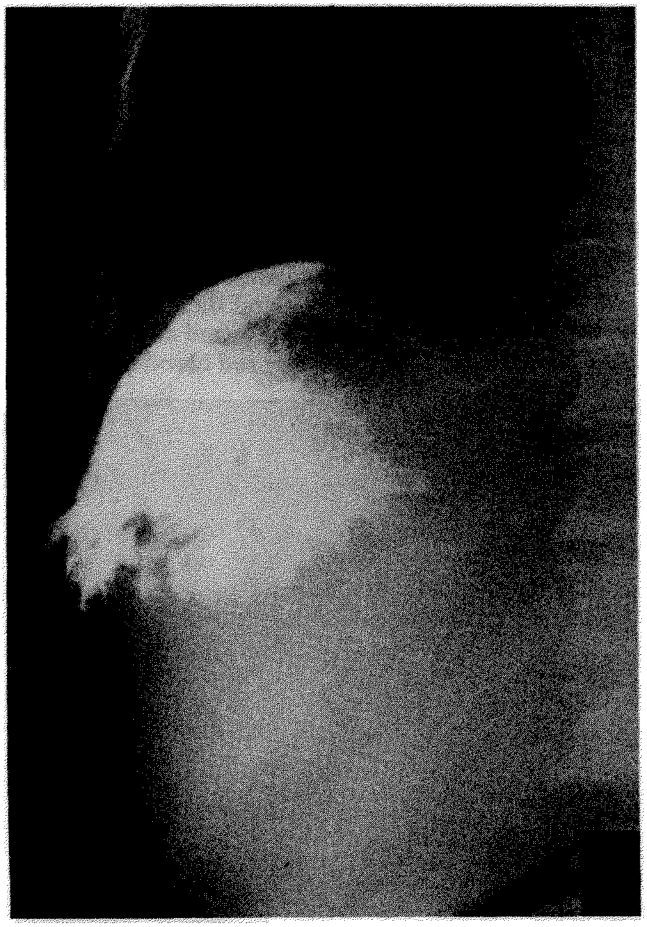

**Figure 2** A contrast roentogenography through PTAD tube in the Case 2 patient. Contrast medium flowed out into the right subphrenic space.

test was positive (1:2048) for *E. histolytica*. Oral adminstration of metronidazole (750 mg/day for 10 days) was effective, and the CT scan showed a reduction in size of the abscess (Figure 4). The PTAD tube was removed on March 19, 1988. The patient was discharged 4 months later and is doing well at this writing (18 months).

### DISCUSSION

Amebic liver abscess is a serious medical problem in areas where amebiasis is endemic, because it is a common complication of amebiasis and is present in over 90% of fatal cases of amebiasis[9]. Even after a correct diagnosis had been made in our two patients, the exact routes of infection remained unknown, however we speculate that these men were asymptomatic *E. histolytica* cyst carriers. Since the

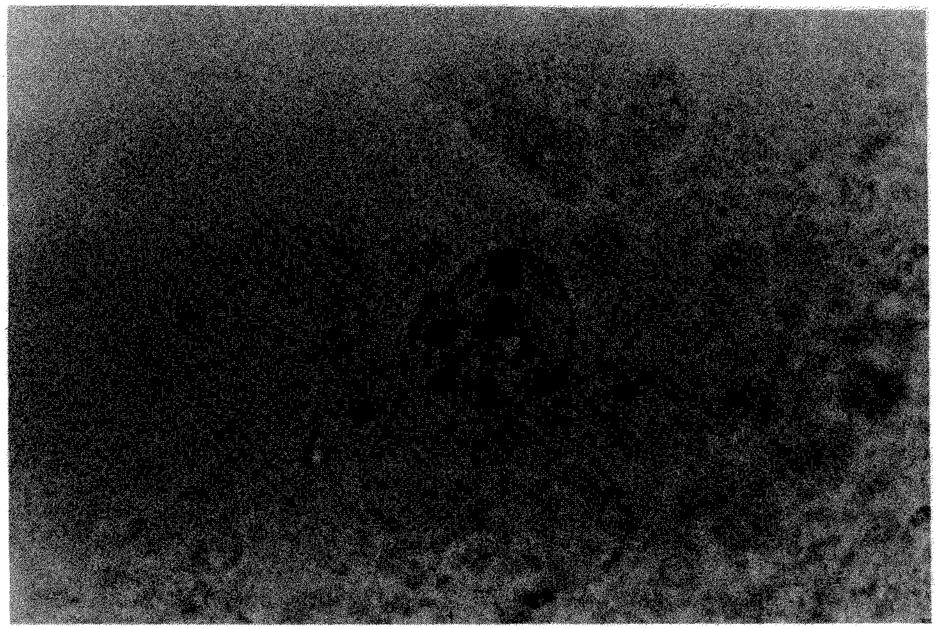

**Figure 3** *Entamoeba histolytica* in material from PTAD tube, phagocyting red blood cells in the Case 2 patient.

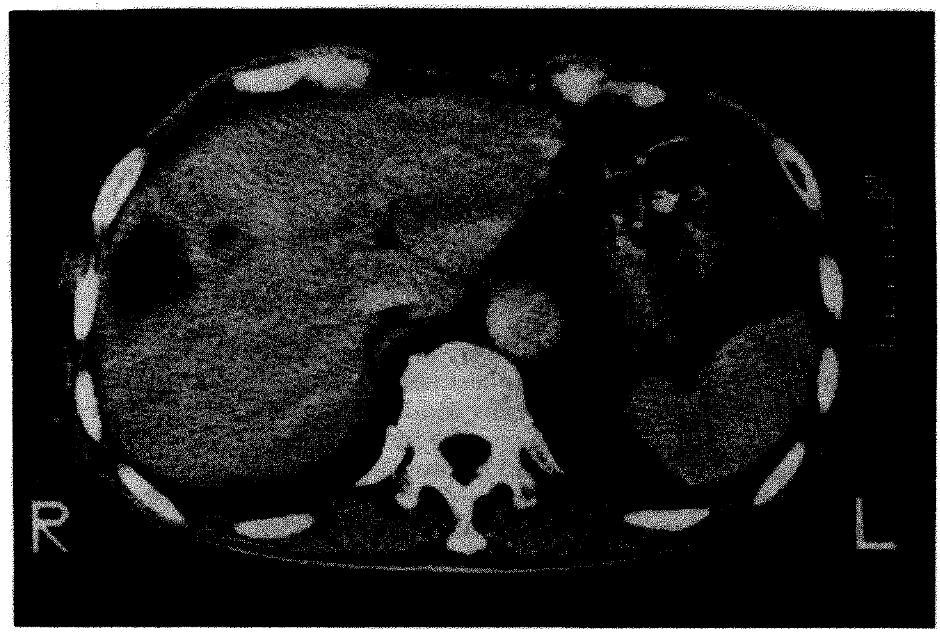

**Figure 4** Computed tomography of amebic liver abscess after metronidazole treatment for the Case 2 patient.

pathogenic potential of *E. histolytica* can be affected by the presence of bacterial cells and/or viruses[10], events which altered the virulence of *E. histolytica* may have occurred. Laboratory data and clinical course suggested a highly virulent infection in the first patients compared to the second one.

Liver abscess can be readily detected by US, CT, scintigraphy, and it is treatable. The differential diagnosis of amebic liver abscess includes hepatic neoplasm, hydatid cyst, and pyogenic liver abscess. Neoplasm can usually be differentiated by ultrasonographic features and laboratory data, and an epidemiologic history is helpful in echinococcosis. One of the most difficult problems is the exclusion of pyogenic liver abscess. Ultrasonographic features which aid in distinguishing amebic liver abscess from pyogenic ones are: 1) round or oval shape, 2) lower echogenicity than the normal liver with internal homogeneous echoes on the high-gain scan[11]. Cultures of the abscess yield pathogens in case of a pyogenic abscess. Immunologic tests for *E. histolytica* antigens[12] and antibodies are useful in excluding pyogenic liver abscess. Positive antibodies indicate an acute amebic infection in areas where the prevalence of amebiasis is low. A history of colitis or bloody diarrhea is often absent in those with an amebic liver abscess, yet the absence of this history does not exclude amebic liver abscess. The most common serious error in detecting and managing amebic liver abscess is failure to consider this disease entity.

Complications of amebic liver abscess are pleural effusion, peritonitis, subphrenic abscess, perforation of the diaphragm, lung abscess, amebic pericarditis and so on. The case-fatality rate is high when the pericardium is involved[8]. In contrast, when the pleurae and lungs are involved, the prognosis is relatively good, probably because hepatobronchial fistula provides a spontaneous drainage. Peritonitis secondary to amebic liver abscess rupture has been reported in 2.4 – 13% of cases and the mortality rate was 40–50%[5,6]. However, the mortality rate was significantly higher in patients with rupture of an amebic liver abscess in whom amebiasis was not suspected[5]. The role of therapeutic aspiration and/or drainage of the abscess cavity and peritoneal space in the managment of ruptured amebic liver abscess has been controversial, and resuscitation, antibiotics, antiamebic agents have improved the surgical results. In our first case, appropriate investigations at the first time of the pleural tap may have led to an early diagnosis of amebic infection. For the second patient with an amebic liver abscess, clinical management was effective, probably because drainage of the abscess cavity and subphrenic space was performed immediately.

Thus, the improved awareness and early diagnosis of this disease entity are the most important factors in achieving a decrease of the morbidity and mortality of amebic liver abscess. In Japan where the incidence of amebic liver abscess is low, one tends not to consider it. From our experience, we would like to stress that physicians should always keep in mind the possibility of amebic liver abscess even in patients who have never been to areas where amebiasis is endemic. A history of colitis or bloody diarrhea, ultrasonographic findings of liver abscess, and immunologic tests aid in the diagnosis of amebic liver abscess. Drainage of the abscess cavity, administration of an amebicidal agent, and surgical drainage of the peritoneal cavity can lead to a favorable result, even when the amebic liver abscess has ruptured.

*Acknowledgements*

We thank T. Akama for technical supports and M. Ohara for reading the manuscript.

*References*

1. William, D.C., Shookhoff, H.B., Felman, Y.M. and Deramos, S.W. (1978) High rates of enteric protozoal infections in selected homosexual men attending a venereal disease clinic. *Sex. Transm. Dis.*, **5**, 155–157
2. Markell, E.K., Havens, R.F., Kuritsubo, B.A. and Wingerd, J. (1984) Intestinal protozoa in homosexual men of the San Francisco bay area: Prevalence and correlates of infection. *Am. J. Trop. Med. Hyg.*, **33**, 239–245
3. Barbour, G.L. and Juniper, K. (1972) A clinical comparison of amebic and pyogenic abscess of the liver in sixty-six patients. *Am. J. Med.*, **53**, 323–334
4. Anderson, W.A.D. (1961) Amebic abscess of the liver. In Pathology, 4th edn. edited by Anderson, W.A.D., pp. 355. St. Louis: Mosby
5. Eggleston, F.C., Handa, A.K. and Verghese, W.D. (1982) Amebic peritonitis secondary to amebic liver abscess. *Surgery*, **91**, 46–48
6. Crane, P.S., Lee, Y.T. and Seel, D.J. (1972) Experience in the treatment of two hundred patients with amebic abscess of liver in Korea. *Am. J. Surg.* **123**, 332–337
7. Adams, E.B. and MacLeod, I.N. (1977) Invasive amebiasis I. Amebic dysentery and its complications. *Medicine*, **56**, 315–323
8. Adams, E.B. and MacLeod, I.N. (1977) Invasive amebiasis II. Amebic liver abscess and its complications. *Medicine*, **56**, 325--334
9. Brandt, H. and Tamayo, R.P. (1970) Pathology of human amebiasis. *Hum. Pathol.*, **1**, 351–385
10. Burchard, G.D. and Mirelman, D. (1988) *Entamoeba histolytica*: Virulence potential and sensitivity to metronidazole and emetine of four isolates possessing nonpathogenic zymodemes. *Exptl. Pathol.*, **66**, 231–242
11. Ralls, P.W., Barnes, P.F., Radin, D.R., Colleti, P. and Halls, J. (1987) Sonographic features of amebic and pyogenic liver abscess: A blinded comparison. *AJR*, **149**, 499–501
12. Gandhi, B.M., Irshad, M., Acharya, S.K. and Tandon, B.N. (1988) Amebic liver abscess and circulating immune complexes of *Entamoeba histolytica* proteins. *Am. J. Trop. Med. Hyg.*, **39**, 440–444

(*Accepted by S. Bengmark on 8 January 1990*)

### INVITED COMMENTARY

The authors present two cases of intra-peritoneal rupture of an hepatic amoebic abscess. One of these patients died. Although on a global scale ameobic abscess is more common than pyogenic abscess, this condition is still uncommon in the West, the exponential growth of international travel however requires a widespread awareness of this condition and these authors draw attention to survival of the second case being associated with rapid diagnosis and appropriate treatment.

The first case was initially diagnosed by ultrasound as cholecystitis. No comment is made as to why this investigation did not demonstrate the abscess in the liver, neither was it diagnosed by the subsequent ultrasound when the patient deteriorated at the referral hospital. In contrast, the second patient was correctly diagnosed by ultrasound although the rupture was only apparent on contrast radiography, at the time of the percutaneous drainage. Metronidazole was life saving and laparotomy was not performed.

There are several lessions to learn from this report. Liver abscess should be considered in patients who are ill with right upper quadrant pain and especially amoebic abscess in patients who have visited areas where amoeba are found. Ultrasound should examine fully the liver as well as the gall bladder. Percutaneous drainage fluid from a collection should be examined for *Entamoeba histolytica*[1]. Stools should also be examined. Rupture of cysts requires urgent surgical toilet of the contaminated area, be it plural, pericardial or peritoneal. Subsequent drainage and administration of metronidazole for an amoebic abscess or other antibiotics for pyogenic abscess is the treatment of choice.

Serological tests, although of limited usefulness for diagnosis of intestinal amoebiases are of some value for the diagnosis of hepatic disease[2].

*References*

1. Ken, J.G., Van Sonnenberg, E. and Casola, G. *et al.* (1989) Perforated amoebic liver abscesses: successful percutaneous treatment. *Radiology*, **170**, 195–197
2. Shetty, N., Das, P., Pal, S.C. and Praphu, T. (1988) Observations on the interpretation of amoebic serology in endemic areas. *J. Trop. Med. Hyg.* **91**, 222–227

Malcolm C.A. Puntis
University of Wales College of Medicine
Cardiff

